# Sacral Extradural Granular Cell Tumor: A Case Report With Electron Microscopy Findings

**DOI:** 10.7759/cureus.100891

**Published:** 2026-01-06

**Authors:** Jaymin Gupta, Madhusudhan Nagesh, Manish Beniwal, Aditi Goyal, Hemnath Elumalai, Yasha T C

**Affiliations:** 1 Neurosurgery, National Institute of Mental Health and Neurosciences, Bengaluru, IND; 2 Neuropathology, National Institute of Mental Health and Neurosciences, Bengaluru, IND

**Keywords:** electron microscopy, extradural, granular cell tumor, peripheral nerve tumor, sacral spine

## Abstract

Granular cell tumors (GCTs) are rare neoplasms of Schwann cell origin, characterized by distinctive histopathological features. They most commonly occur in the head, neck, and upper digestive tract and rarely involve peripheral nerves, although immunohistochemistry and electron microscopy have, in some cases, confirmed a neural origin. A 33-year-old male presented with pain and paresthesia of the left lower limb for two years. MRI of the lumbosacral spine revealed an extradural lesion in the left S1 neural foramen. He underwent a left S1 hemilaminectomy and excision of the lesion. Histopathology, supported by immunohistochemistry and electron microscopy, confirmed a diagnosis of GCT. Histological confirmation is critical, as these lesions can radiologically mimic other tumors commonly found in this location, and treatment may need to be modified for more aggressive tumor subtypes.

## Introduction

Granular cell tumors (GCTs), also called Abrikossoff tumors or myoblastomas, are rare neoplasms that typically arise in the skin, oral cavity, upper gastrointestinal tract, and subcutaneous tissue [1]. Although uncommon, occurrences within the central nervous system have also been documented. In the brain, they are usually located in the sella, involving the posterior pituitary, and are benign [2]. Granular cell astrocytomas involve the cerebral hemispheres and are malignant [3]. In the spine, GCTs present as intradural extramedullary tumors compressing the spinal cord, with approximately 20 cases reported in the literature [4], and only two reports of sacral nerve root involvement [5,6]. We report the third case of a GCT involving the left S1 nerve root, with electron microscopy findings.

## Case presentation

A 33-year-old man presented with a two-year history of pain and paresthesia over the posterior aspect of his left leg and the sole of his left foot. The pain was moderate in intensity and worsened with sitting for more than 10-15 minutes, standing, bending forward, and lying on the left side at night. It was also aggravated by strenuous activity and was refractory to pain medications. Neurological examination revealed no focal deficits.

**Figure 1:**
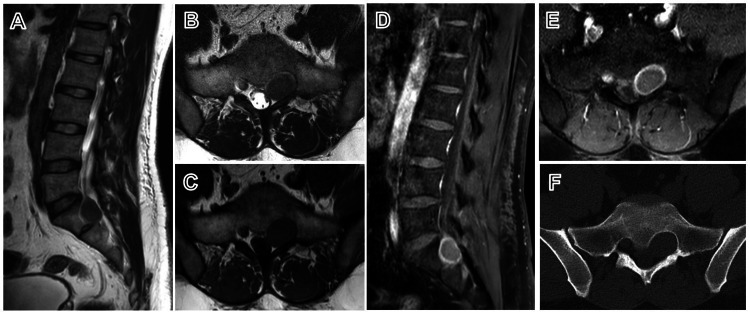
MRI of the lumbosacral spine T2 sagittal (A) and T2 axial (B) images show an isointense lesion in the left S1 neural foramen; T1 axial (C) demonstrates isointensity; T1 contrast sagittal (D) and axial (E) images show peripheral enhancement. CT axial image (F) demonstrates scalloping and widening of the left S1 neural foramen.

MRI of the lumbosacral spine demonstrated a well-defined ovoid lesion along the left S1 root, expanding the neural foramen, measuring 3.6 × 3 × 3.2 cm. The lesion was isointense on both T1- and T2-weighted images and showed peripheral contrast enhancement on T1-weighted sequences. CT of the spine showed scalloping and widening of the left S1 neural foramen without bony destruction, consistent with a slow-growing expansile lesion. No intradural component was identified (Figure 1).

**Figure 2:**
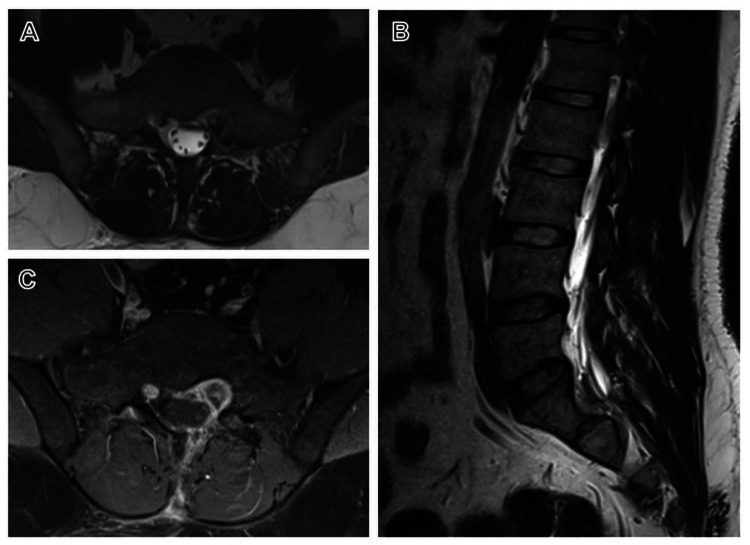
MRI of the lumbosacral spine T2 axial (A) and T2 sagittal (B) images show a small residual lesion in the left S1 foramen. Post-contrast T1 image (C) demonstrates peripheral enhancement of the lesion.

Under C-arm guidance, a left S1 hemilaminectomy was performed. The thecal sac was displaced to the right, and a lesion was identified ventrolateral to it. The lesion had a thick, whitish capsule containing a yellowish, avascular, soft component. The capsule could not be fully dissected from the nerve root; a capsule layer was intentionally left behind to avoid a neurological deficit. Postoperatively, the patient reported improvement in pain and paresthesia, with no new neurological deficits. At one-year follow-up, he was symptom-free, and MRI demonstrated a stable, small residual lesion (Figure 2).

Histopathological examination revealed a tumor composed of polygonal cells arranged in nests and sheets. Many cells exhibited indistinct cell membranes. The tumor cells had round nuclei with fine chromatin and small, conspicuous nucleoli. The cytoplasm was abundant, eosinophilic, and prominently granular. Mitoses were rare. Both the tumor and adjacent soft tissue showed a prominent lymphoplasmacytic infiltrate. Focally, the tumor exhibited necrosis and infiltration into nerves and ganglia.

**Figure 3:**
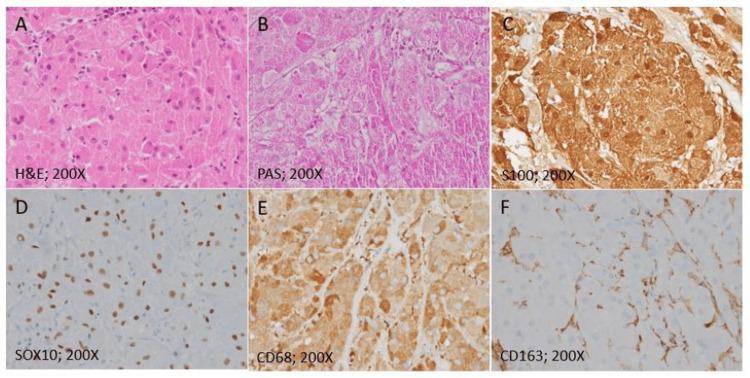
Histopathology and immunohistochemistry of the GCT (A) Tumor composed of polygonal cells with indistinct cell membranes, round nuclei, fine chromatin, and small, conspicuous nucleoli. The cells have abundant eosinophilic, prominently granular cytoplasm. (B) PAS staining highlights cytoplasmic granules within tumor cells. Immunohistochemistry demonstrates tumor cell positivity for S100 (C), SOX10 (D), and CD68 (E), and negativity for CD163 (F). GCT, granular cell tumor; PAS, Periodic Acid-Schiff

Periodic Acid-Schiff (PAS) staining highlighted cytoplasmic granules within the tumor cells. Immunohistochemistry revealed positivity for SOX10, CD68, and S100, and negativity for synaptophysin, chromogranin, neurofilament, and EMA. Ki-67 labeling showed occasional tumor cells and numerous inflammatory cells. These findings were consistent with a benign GCT (Figure 3).

**Figure 4:**
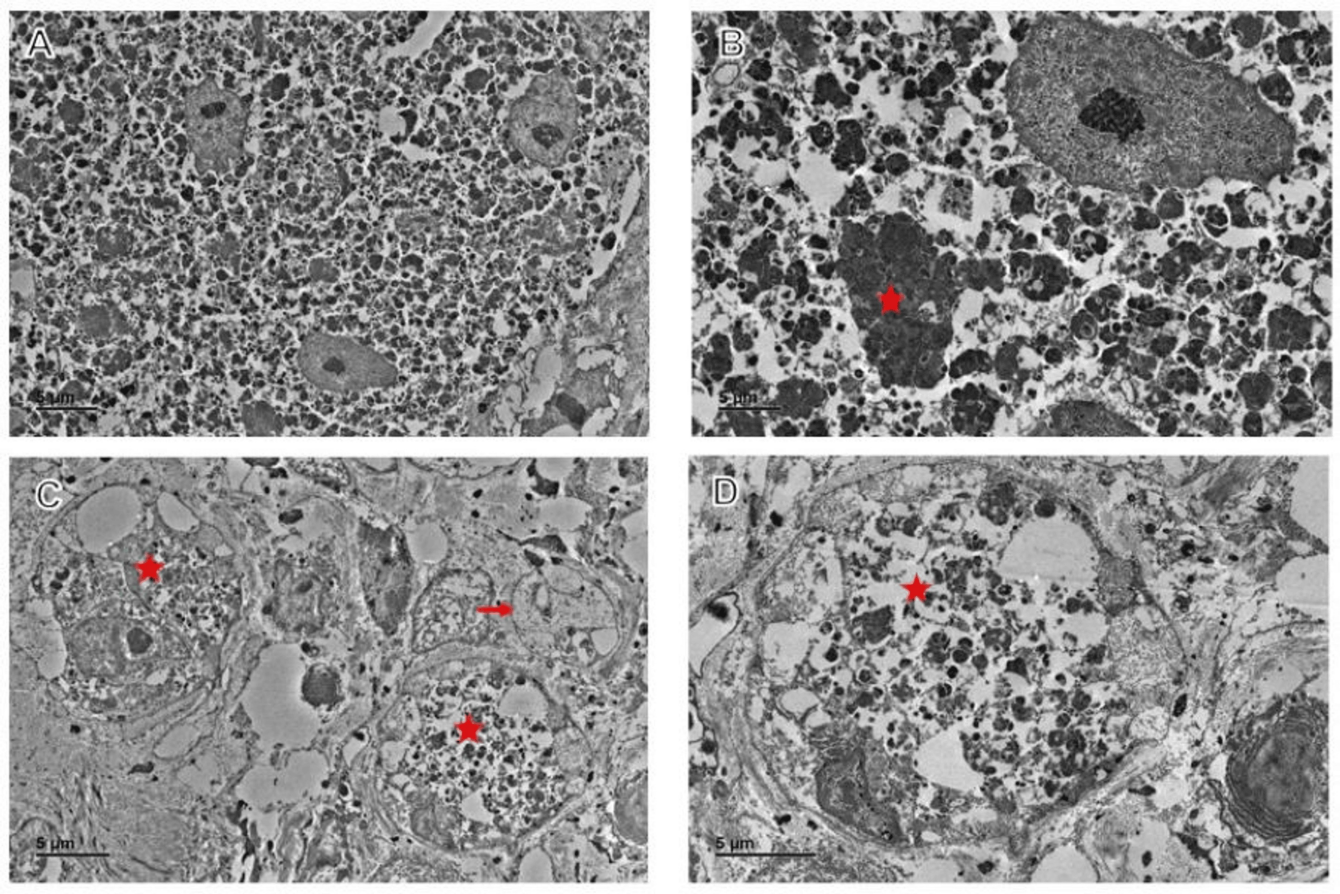
Electron micrographs of the GCT (A) Group of tightly apposed dorsal root ganglion cells. (B) Numerous cytoplasmic accumulations of pleomorphic, heterogeneous, osmiophilic material (star). (C) Low magnification of dorsal root ganglion cells (star) filled with heterogeneous, osmiophilic material; arrow indicates an unmyelinated nerve fiber. (D) High magnification of a dorsal root ganglion cell filled with pleomorphic, heterogeneous, osmiophilic material (star). GCT, granular cell tumor

Electron microscopy showed a group of tightly apposed dorsal root ganglion cells with numerous cytoplasmic accumulations of pleomorphic, heterogeneous, osmiophilic material (Figure 4).

## Discussion

GCTs are peripheral nerve sheath tumors exhibiting neuroectodermal differentiation. These tumors are thought to arise from Schwann cells, as indicated by immunohistochemical and electron microscopy findings [7]. Although they originate from Schwann cells, spinal canal GCTs are exceedingly rare. Most spinal GCTs are located in the intradural extramedullary compartment [4], with a few reports suggesting a possible intramedullary location as well [8]. Our case involved an extradural GCT along the sacral nerve root, with only two prior cases reported [5,6]. Another case of extradural GCT has been reported at the thoracic level, where the patient presented with spinal deformity [9].

MRI with contrast is the imaging modality of choice. GCTs appear hypo- to isointense on T1- and T2-weighted sequences and demonstrate heterogeneous enhancement following contrast administration. These MRI characteristics are nonspecific and may overlap with other common differential diagnoses for extradural lesions along the neural foramina, such as neurofibroma or schwannoma. Therefore, histopathological confirmation is imperative.

GCTs typically exhibit infiltrative, nonencapsulated growth patterns, forming nests, cords, or sheets of polygonal and occasionally spindle-shaped cells with abundant, finely granular, eosinophilic cytoplasm. These cytoplasmic granules are PAS-positive and diastase-resistant, consistent with a lysosomal origin [10]. Immunohistochemical analysis typically shows positive expression of markers such as S-100, CD68, neuron-specific enolase, CD57, inhibin, calretinin, TFE3, SOX10, CD56, PGP9.5, and vimentin [10]. Electron microscopy demonstrates ovoid or polyhedral tumor cells containing irregular osmophilic granules that vary in size, shape, and density, often arranged in clusters. Occasional unmyelinated axons can be observed among the granular cell clusters.

Schwannoma and neurofibroma are the main differential diagnoses at this anatomical site. Histologically, schwannomas exhibit alternating hypercellular (Antoni A) and hypocellular (Antoni B) areas, with characteristic Verocay bodies. The neoplastic cells are spindle-shaped with elongated, wavy nuclei, and perivascular hyalinization is frequently present. Neurofibromas are composed of interlacing bundles of spindle cells embedded in a myxoid matrix. Notably, cells in both schwannomas and neurofibromas lack the cytoplasmic granularity that is a hallmark of GCTs.

Spinal GCTs are usually benign and are treated with complete surgical excision when feasible. None of the reported spinal GCTs have demonstrated malignancy or metastasis. Recurrence has been reported in a few cases following incomplete excision, and these patients were subsequently treated with radiation therapy [11]. In the report by Mundi et al., total excision was avoided because the nerve root was merging with the tumor capsule [6].

## Conclusions

GCTs are rare peripheral nerve sheath tumors that infrequently involve the spinal canal, where they typically present as intradural extramedullary lesions. Histological confirmation is essential, as these tumors can radiologically mimic other more common lesions in this location.
